# Individual and Combined Effects of *Bacillus Thuringiensis* and Azadirachtin on *Plodia Interpunctella Hübner* (Lepidoptera: Pyralidae)

**DOI:** 10.1093/jisesa/iew086

**Published:** 2016-08-25

**Authors:** Gadir Nouri-Ganbalani, Ehsan Borzoui, Arman Abdolmaleki, Zahra Abedi, Shizuo George Kamita

**Affiliations:** ^1^Department of Plant Protection, Faculty of Agricultural Sciences, University of Mohaghegh Ardabili, Ardabil, Iran; ^3^Department of Entomology and Nematology, College of Agricultural and Environmental Sciences, University of California, Davis, CA

**Keywords:** Azadirachtin, *Bacillus thuringiensis*, bioassay, biorational insecticide, the Indian meal moth

## Abstract

The Indianmeal moth, *Plodia interpunctella* Hübner (Lepidoptera: Pyralidae), is a major stored product pest that is found throughout the world. In this study, the effect of oral exposure to *Bacillus thuringiensis* (Berliner) subsp. *kurstaki* (Bacillales: Bacillaceae) and azadirachtin was evaluated in third instar *P. interpunctella* under laboratory conditions. The median lethal concentration (LC_50_) of *Bt* and azadirachtin on third instars was 490 and 241 μg a.i./ml, respectively. The median lethal time (LT_50_) of these insecticides was the same (4.5 d following exposure to 750 or 400 μg a.i./ml of *Bt* or azadirachtin, respectively). When the larvae fed on diet containing LC_30_ concentrations of both *Bt* and azadirachtin an additive interaction in terms of mortality was found. A synergistic interaction was found when the larvae fed on diet containing LC_50_ concentrations of both insecticides. Larvae that fed on insecticide-containing diet (either *Bt* or azadirachtin at an LC_30_ concentration, or both insecticides at LC_30_ or LC_50_ concentrations) showed lower glycogen and lipid levels, and generally lower protein content in comparison to control larvae. Larvae that fed on diet containing both *Bt* and azadirachtin showed reduced weight gain and nutritional indices in comparison to control larvae or larvae fed on the diet containing only one of the insecticides. Finally, exposure to both insecticides, either individually or in combination, reduced the level of digestive enzymes found in the midgut. Our findings indicate that both *Bt* and azadirachtin, either individually or in combination have significant potential for use in controlling of *P. interpunctella*.

The Indian meal moth, *Plodia interpunctella* Hübner (Lepidoptera: Pyralidae), is a widespread and serious insect pest of stored products including nuts, bulk-stored grain, and dried fruits (Johnson et al. 1992, Doud and Phillips 2000, Nansen and Phillips 2004). Infestation of stored products by this pest can inflict severe economic losses due to direct feeding damage and indirectly through reduced quality (Phillips et al. 2000). The present methods for control of this pest depend mainly on the extensive use of synthetic chemical insecticides (Mullen and Dowdy 2001). Methyl bromide and phosphine, fumigants that were historically used for the control of stored product pests, are now prohibited in most developed countries. Also, there is evidence that *P. interpunctella* has developed resistance to phosphine (Zettler et al. 1989, Chaudhry 1997, Mohandass et al. 2007). For these reasons, there is a need to develop alternative and environmentally friendly insecticides for the control of *P. interpunctella*. Since *P. interpunctella* is an external feeder; surface insecticidal treatments by *Bacillus thuringiensis* (Berliner) subsp. *kurstaki* (Bacillales: Bacillaceae) and azadirachtin have been advocated as biopesticide alternatives or supplements for the integrated management of this pest (Phillips et al. 2000, Arthur and Phillips 2003).

*B. thuringiensis* (*Bt*) is a widespread, spore-forming soil bacterium. Some *Bt* strains produce proteinaceous crystals (δ-endotoxins) during sporulation that are highly toxic to insects (Navon 2000, Gajendra Babu et al. 2002, Ibargutxi et al. 2008). Following ingestion by larval insects the crystals are solubilized in the midgut and subsequently activated by proteases. The activated toxins bind to midgut epithelial cells; form pores, and cause osmotic cell lysis eventually leading to insect death. Several *Bt* toxins and *Bt* toxin genes have been commercially developed for the control of pest insects found in Lepidoptera, Diptera, and other insect orders.

Azadirachtin is an insecticidal compound that was originally isolated from the seeds of the neem plant *Azadirachta indica* (Mordue and Blackwell 1993, Isman 2006). Azadirachtin induces several effects in insects such as growth disruption, feeding and oviposition deterrence, and reductions in fitness and fecundity (Mancebo et al. 2002, Defagó et al. 2011). This tetranotriterpenoid compound is considered generally safe to beneficial insects (Condor Golec 2007) and shows high potential for the integrated management of noxious pest insects.

The individual effects of *Bt* and azadirachtin have been well studied on numerous pest insects (Gajendra Babu et al. 2002, Mancebo et al. 2002, Senthil Nathan et al. 2004, Zhu et al. 2007, Railda Roel et al. 2010). However, there are relatively few studies (e.g., Abedi et al. 2014) on the combined effects of *Bt* and azadirachtin. Here, we study the effects of *Bt* and azadirachtin on third-instar larvae of *P. interpunctella*. Specifically, we assess mortality, weight, nutritional indices, energy reserves, and digestive enzyme activity in larvae that fed on artificial diet containing lethal or sublethal concentrations of *Bt* and azadirachtin either individually or in combination.

## Materials and Methods

### Insect Culture

Indian meal moth larvae were collected in May 2015 from stored walnut fruit containers in Sabzevar, Iran. About 60 larvae were transferred to artificial diet composed of wheat bran (800 g), brewer’s yeast (160 g), honey (200 ml), and glycerin (200 ml), and reared at 27 ± 1 °C, 65 ± 5% RH, and a photoperiod of 16:8 (L:D) h. The larvae were placed in 1.5 L plastic containers with 200 g of artificial diet. The adults that emerged were removed and allowed to mate in a new 1.5 liters plastic container. Following oviposition the eggs were allowed to develop at their oviposition sites under the standard rearing conditions described earlier. The larvae that emerged were allowed to feed on fresh artificial diet. The insects were reared for three generations under these standard rearing conditions. Under these conditions, *P. interpunctella* showed five larval instars with a life-cycle lasting 23–28 d.

### Insecticides

Commercial formulations of azadirachtin, NeemGuard 1% EC (Shalimar International LLC Co., Dubai, UAE), and *Bt* subsp. *kurstaki*, Rapax 7.5% EC (Intrachem Bio, Grassobio BG, Italy), were used in this study.

### Individual Effects of *Bt* and Azadirachtin on the Mortality of *P. interpunctella*

In order to determine the lethal effects of *Bt* and azadirachtin on *P. interpunctella*, newly emerged third-instar larvae were allowed to feed on artificial diet that was mixed with *Bt* or azadirachtin. Initial dose-response experiments were carried out with the following insecticide concentrations: 750, 610, 496, 394, 320, and 225 μg a.i./ml for *Bt*; and 400, 302, 230, 174, 132, and 100 μg a.i./ml for azadirachtin. Stock dilutions of the insecticides were made with distilled water containing 200 ppm of Tween 80 (Merck, Darmstadt, Germany) as a surfactant (Rosenheim and Hoy 1988). In order to prepare the insecticide containing diet, 1 ml from each insecticide stock dilution was mixed into 9 g of the artificial diet as described previously in Abedi et al. (2014). Control artificial diet was prepared with distilled water containing 200 ppm of Tween 80. In order to induce a higher feeding rate, the third-instar larvae were starved for 12 h prior to insecticide exposure (Ibargutxi et al. 2008). Following starvation, groups of 15 larvae were transferred into a sterile Petri dish (8 cm in diameter × 2 cm height) containing 10 g of insecticide-containing diet or control diet. The Petri dishes were placed in a growth chamber that was set at the above described standard rearing conditions. The larvae were allowed to feed on the insecticide-containing or control diet for the duration of the bioassay. Larval mortality was recorded at 24 h intervals for 5 d. Each concentration was replicated three times, and 15 adult females were used in each insecticide concentration. The bioassay trials were repeated three times.

The median effective time to cause 50% mortality of third instar *P. interpunctella* was determined using larvae that fed on diet (9 g) that was mixed with 1 ml of insecticide stock (750 and 400 μg a.i./ml for *Bt* and azadirachtin, respectively) as described previously in Saber and Abedi (2013). Larval mortality was recorded at 24 h intervals for 5 d. Each concentration was replicated three times, and 15 adult females were used in each insecticide concentration. The bioassay trials were repeated three times.

### Combined Effects of *Bt* and Azadirachtin on the Mortality of *P. interpunctella*

In order to determine the lethal effects of exposure to both *Bt* and azadirachtin, newly molted third-instar larvae of *P. interpunctella* were allowed to feed on diet containing LC_30_ concentrations of both *Bt* and azadirachtin or LC_50_ concentrations of both insecticides. Artificial diet containing both *Bt* and azadirachtin was prepared as described earlier except that both insecticides were diluted in 1 ml of distilled water containing 200 ppm of Tween 80. Control insects were allowed to feed on diet containing an LC_30_ or LC_50_ concentration of each insecticide individually. These diets were prepared as described earlier. The larvae were exposed to the insecticide containing diet and reared as described earlier. Each treatment group consisted of 15 larvae. Mortality was scored at 24 h intervals for 5 d. The experiments were performed in triplicate for each insecticide concentration and each bioassay was repeated three times.

## Biochemical Analyses

For the biochemical analyses, newly molted third-instar larvae were allowed to feed on diet containing *Bt* and azadirachtin (individually or in combination) as well as control diet as described earlier. After 5 d of feeding on these diets, larvae from each treatment were individually cold-anesthetized at 5 °C. Subsequently, total protein, glycogen content, and lipid content were determined from insecticide-exposed and control larvae as described below. Six to eight replicates were performed for each treatment.

## Protein Determination

Protein concentration was determined by the procedure of Lowry et al. (1951). In this method, the whole larva was homogenized on ice in 1 ml of 50 mM sodium phosphate buffer (pH 7) in a 1.5 ml microcentrifuge tube using a pre-cooled Teflon pestle. Following the homogenization, 20 µl of the homogenate was added to 100 µl of Lowry reagent (Ziest Chem. Co., Tehran, Iran) and the mixture was incubated for 30 min at room temperature prior to absorbance measurement at 545 nm. Bovine serum albumin was used as a protein concentration standard.

## Glycogen Determination

Glycogen content was estimated using anthrone reagent as described by Yuval et al. (1998) with some modifications. Each larva was individually homogenized as described earlier in 1 ml of ethanol saturated with sodium sulphate. The homogenate was centrifuged at 12,000 *g* for 10 min at 4°C and the supernatant discarded. The pellet was resuspended by vigorous shaking in 0.5 ml of 70% ethanol. The suspension was then centrifuged as described above and the final pellet was heated to remove residual ethanol. The dried residue was dissolved in 0.5 ml of 30% potassium hydroxide and placed in a boiling water bath for 15 min. After cooling to room temperature, 1 ml of absolute ethanol was added, and the solution was centrifuged again as described earlier. Following centrifugation, the supernatant was carefully collected and pooled. The pellet was dissolved in 0.5 ml of distilled water, and then 2 ml of anthrone reagent (0.05% in sulphuric acid) was added to the solution. After mixing, the mixture was heated for 15 min at 90 °C and then cooled to room temperature. The absorbance of the solution was read at 620 nm, and glycogen content was quantified by comparison to a glycogen standard curve.

## Lipid Determination

Lipids were extracted from individual larvae by the method of Van Handel (1985) with some modifications. Each larva was homogenized in 1 ml of chloroform-methanol (1:1 v/v) as described earlier, and the homogenate was centrifuged at 2,000 *g* for 10 min at 4 °C. The supernatant was then mixed with 1.5 ml of chloroform-distilled water (2:1 v/v) and centrifuged at 3,000 *g* for 1 h at 4 °C; and the aqueous phase was discarded. This liquid–liquid extraction was repeated twice, and the lipid extract was evaporated to dryness. Lipid content was quantified by the method of Yuval et al. (1998). The extract was incubated with 1 ml of sulphuric acid for 10 min at 100 °C, and then cooled to room temperature, and 5 ml of sulphosphovanillin reagent (orthophosphoric acid 0.6% aqueous vanillin solution 4:1 v/v) was added. After 40 min at room temperature, the absorbance of the solution was read at 530 nm. The lipid content was determined by comparison to a standard curve that was prepared using cholesterol palmitate.

## Midgut Homogenate Preparation and Digestive Enzyme Activity Assays

Third-instar larvae of *P. interpunctella* were allowed to feed on diet containing an LC_30_ concentration of *Bt* or azadirachtin or LC_30_ concentrations of both *Bt* and azadirachtin or LC_50_ concentrations of both *Bt* and azadirachtin, and then reared as described above. After 5 d of insecticide exposure, the surviving larvae were individually submerged in ice-cold 0.15 M NaCl and the midguts were dissected with the aid of a stereomicroscope (Stemi SV6 ZEISS, Germany) following the method of Borzoui and Bandani (2013). For each treatment, 50 midguts were dissected and then transferred into 1 ml of 10 mM NaCl. The dissected midguts with their lumen content were then homogenized using a precooled Teflon pestle and the homogenate was centrifuged at 15,000 *g* for 15 min at 4 °C. The supernatant was stored at −20 °C prior to use as an enzyme source for the activity assays. α-Amylase activity was determined by the method of Bernfeld (1955) using 1% starch as a substrate in Tris-HCl buffer (pH 8). General proteolysis activity was determined by the method of Elpidina et al. (2001) using 1.5% azocasein as a substrate in glycine-NaOH buffer (pH 10).

## Determination of Nutritional Indices Following Insecticide Exposure

In order to explore the long-term effects of exposure to *Bt* and azadirachtin, nutritional indices were determined as described by Farrar et al. (1989), Manuwoto and Scriber (1982), and Waldbauer (1968) with some modifications as described by Huang et al. (1997). For these experiments, newly emerged third instar *P. interpunctella* were allowed to feed on artificial diet that was mixed with LC_30_ and LC_50_ stock concentrations of *Bt* and azadirachtin as described in Sections “Individual effects of *Bt* and azadirachtin on the mortality of *P. interpunctella*” and “Combined effects of *Bt* and azadirachtin on the mortality of *P. interpunctella*”. Groups of 20 third-instar larvae were allowed to feed on 10 g of the insecticide containing diet or control diet that was placed in a Petri dish. One replicate consisted of 5 groups of 20 larvae, and five replicates were performed for each treatment. The initial mass of the control and treated larvae was measured individually before the larvae were transferred onto the diet. After 5 d of feeding, the mass of each surviving larvae (control and experimental) was again determined. The mass of the fecal pellets (following oven drying for 48 h at 60 °C) was also determined. The dry weight of the artificial diet (10 g wet weight) and larvae (20 randomly chosen larvae) was determined after they were oven-dried for 48 h at 60 °C. The nutritional indices of the larval *P. interpunctella* were calculated using the formulae described by Farrar et al. (1989), Manuwoto and Scriber (1982), and Waldbauer (1968) as follows:

Approximate digestibility (AD) = E − F/E; Consumption index (CI) = E/A; Efficiency of conversion of ingested food (ECI) = P/E; Efficiency of conversion of digested food (ECD) = P/E − F; Relative consumption rate (RCR) = E/A × T; Relative growth rate (RGR) = P/A × T. where, A = mean dry weight of larvae over the feeding period (mg), E = dry weight of food consumed (mg), F = dry weight of feces produced (mg), P = dry weight gain of larvae (mg), and T = duration of feeding period (day).

## Data Analysis

The result of each trial was tested for curve fit using PROC GENMOD procedures (SAS Institute 2002; Robertson et al. 2007), and the data were analyzed using PROC PROBIT in order to determine lethal concentrations (LC_30_, LC_50_, and LC_90_) on standard and log scales with associated 95% fiducial limits (FLs). In addition, the mean mortality of *P. interpunctella* that fed on different concentrations of insecticide was analyzed by ANOVA with mean separation at a 5% level of significance by LSD testing. The data for LT_50_ determination were analyzed using SAS software.

The mortality data were corrected using Abbott’s formula (Abbott, 1925). Then the expected mortality (ME) for the combination of azadirachtin with *Bt* was calculated through formula ME = MC + MM (1 − MC), MC: mortality caused by either azadirachtin; MM: observed mortality caused by *Bt*. Then chi-square (χ^2^) values were calculated by formula (MCM − ME)^2^/ME; where MCM is the observed mortality for the combination of azadirachtin and *Bt*. If the calculated chi-square was > 3.89 (as specified for df = 1), a non-additive effect of the two control agents was indicated. The difference MCM − ME > 0 indicated synergism; and the difference MCM − ME < 0 indicated antagonism (Koppenhöfer and Kaya, 1996).

Data regarding energy reserves, nutritional indices, and digestive enzyme activity were analyzed by ANOVA with mean separation at a 5% level of significance by LSD testing using SAS software.

## Results

### Mortality of *P. interpunctella* Following Insecticide Exposure

Third-instar larvae of *P. interpunctella* were susceptible to *Bt* and azadirachtin that were incorporated into the diet. The LC_30_, LC_50_, and LC_90_ concentrations of these insecticides are given in Table 1. The LC_30_ and LC_90_ concentrations of *Bt* were from 1.5 to 2.2 times higher than those of azadirachtin. The LT_50_ values of *Bt* and azadirachtin in third instar *P. interpunctella* are shown in Table 2.

**Table 1:** Toxicity of ***Bt*** and azadirachtin on third-instar larvae of *P. interpunctella*

Insecticide	***n***^*a*^	χ^2^	Slope ± SE	**Lethal concentration (µg a.i./ml)**		
				LC_30_ (95% FL)	LC_50_ (95%FL)	LC_90_ (95%FL)
***Bt***	315	36.61	3.34 ± 0.55	341.7	490.4	1,186
				(279.9–388.9)	(435.4–560.6)	(920.2–1,907)
**azadirachtin**	315	37.44	2.77 ± 0.45	155.9	241.1	700.2
				(124.5–181.5)	(209.1–285.6)	(512.1–1,255)

Lethal concentrations and 95% FLs were estimated using logistic regression (SAS Institute 2002).

^*a*^ The total number of larval used for bioassay test.

**Table 2:** Lethal time of *Bt* and azadirachtin in third-instar larvae of *P. interpunctella*

Insecticide	Insecticide concentration (µg a.i./ml)		χ^2^	Slope ±SE	**Lethal time (day)**		
		**n**^*a*^			LT_30_ (day) (95% FL)	LT_50_ (day) (95%FL)	LT_90_ (day) (95%FL)
***Bt***	750	45	17.8	15.7 ± 3.7	4.2 (3.8–4.4)	4.5 (4.3–4.8)	5.5 (5.1–6.5)
**azadirachtin**	400	45	14.9	14.5 ± 3.7	4.16 (3.7–4.4)	4.5 (4.3–4.8)	5.5 (5.1–6.9)

Lethal times and 95% FL were estimated using logistic regression (SAS Institute 2002).

^*a*^ The n value shows the sample size for each parameter.

**Fig. 1.**
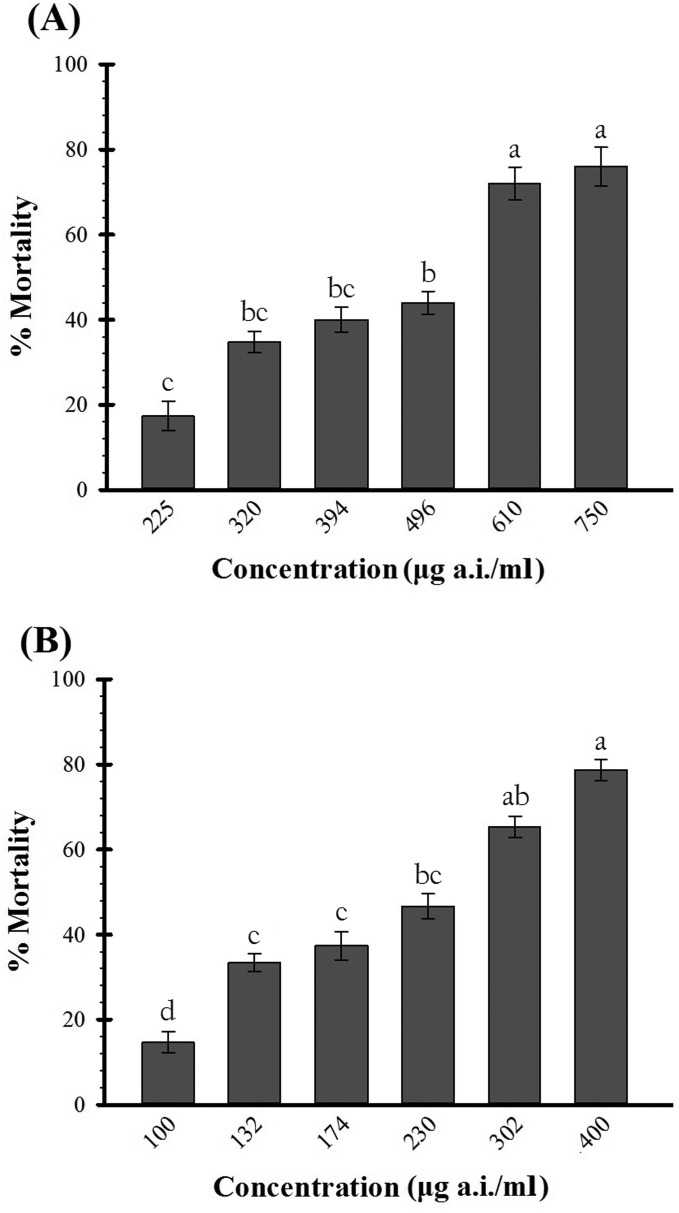
Mean (±SE) percentage mortality (*n* = 5) of third instar *P. interpunctella* exposed to different concentrations of *Bt* (A) or azadirachtin (B). Larval mortality was recorded after 5 d of continuous exposure to the insecticide. The error bars indicate standard error of the mean of three replicate experiments. The lowercase letters above the bars indicate statistically significant differences between values (LSD test, *P* < 0.05).

After 5 d of insecticide exposure, the percentage mortality increased with increasing concentrations of *Bt* (*F*_5, 12_ = 18.74, *P* < 0.001) or azadirachtin (*F*_5, 12_ = 29.54, *P* 0.001). With the concentration ranges that were tested in this study, a mortality range of ∼20% at the lowest dose and 80% at the highest dose was obtained for both *Bt* and azadirachtin (Fig. 1).

### Interaction Between *Bt* and Azadirachtin

Third instar *P*. *interpunctella* that were fed diet containing LC_30_ concentrations of both *Bt* and azadirachtin or LC_50_ concentrations of both insecticides showed additive and synergistic interactions, respectively (Table 3).

**Table 3:** Interaction of *Bt* and azadirachtin insecticides on third-instar larvae of *P. interpunctella*^*a*^

Insecticide	Observed mortality (%)	ME (%)	χ^2^	Interaction^*b*^
***Bt* + azadirachtin (LC_30_)**	50.7	39.4	3.2	additive
***Bt* + azadirachtin (LC_50_)**	73.3	50.4	10.4	synergistic

^*a*^ The larvae were continuously exposed to LC_30_ (341.7 and 155.9 µg a.i./ml, respectively) or LC_50_ (490.4 and 241.1 µg a.i./ml, respectively) concentrations of both *Bt* and azadirachtin for 5 d prior to scoring mortality.

^*b*^ The type of interaction (synergistic, additive or antagonistic) following exposure to both *Bt* and azadirachtin was determined by comparing the expected and observed mortalities as described by Koppenhöfer and Kaya (1996).

### Energy Reserves of *P. interpunctella* Following Insecticide Exposure

Third instar *P. interpunctella* that continuously fed on insecticide containing diet for 5 d showed a statistically significant reduction in energy reserves (*F*_4, 20_ = 37.35, *P*  0.001 for protein; *F*_4, 20_ = 63.68, *P*  0.001 for glycogen and *F*_4, 20_ = 89.39, *P*  0.001 for lipid) in comparison to control larvae (Table 4). Exposure to an LC_30_ concentration of *Bt* resulted in 32, 52, and 32% reductions in protein, glycogen, and lipid content, respectively, in comparison to control larvae. Exposure to an LC_30_ concentration of azadirachtin resulted in similar reductions in glycogen (55%) and lipid (31%) content, however, a statistically significant difference was not found in protein content. Exposure to both *Bt* and azadirachtin (at either LC_30_ or LC_50_ concentrations of each insecticide) resulted in a greater magnitude in the reduction in glycogen content (67 and 78%, respectively) and lipid content (47 and 58%, respectively) in comparison to larvae that fed on diet containing only one of the insecticides. The reduction in protein content (34 and 39%, respectively) of larvae that were fed on diet containing both insecticides (LC_30_ or LC_50_ concentrations of each insecticide), however, was not statistically different than that of larvae that fed on *Bt* containing diet.

**Table 4:** Energy reserves of third-instar larvae of *P. interpunctella* following exposure to *Bt* and azadirachtin^*a*^

Treatment	***n***^*b*^	Protein (µg/larva)	Glycogen (µg/larva)	Lipid (µg/larva)
**Control**	**5**	142.8 ± 3.7a	41.6 ± 2.6a	204.8 ± 6.0a
***Bt* (LC_30_)**	**5**	97.2 ± 4.6b	19.8 ± 1.4b	139.2 ± 5.7b
**azadirachtin (LC_30_)**	**5**	132.6 ± 3.6a	18.6 ± 1.2b	141.2 ± 4.5b
***Bt* + azadirachtin (LC_30_)**	**5**	94.6 ± 3.3b	13.6 ± 1.0c	108.0 ± 3.1c
***Bt* + azadirachtin (LC_50_)**	**5**	87.8 ± 4.9b	9.2 ± 1.0c	86.4 ± 3.8d

^*a*^ The larvae were continuously exposed to LC_30_ (341.7 and 155.9 µg a.i./ml, respectively) and LC_50_ (490.4 and 241.1 µg a.i./ml, respectively) concentrations of *Bt* and azadirachtin for 5 d prior to measuring energy reserves. Mean values followed by different letters in the same column are significantly different (LSD, *P* < 0.05).

^*b*^ The *n* value shows the sample size for each parameter.

### Digestive Enzyme Activity in Midguts from *P. interpunctella* Following Insecticide Exposure

**Fig. 2.**
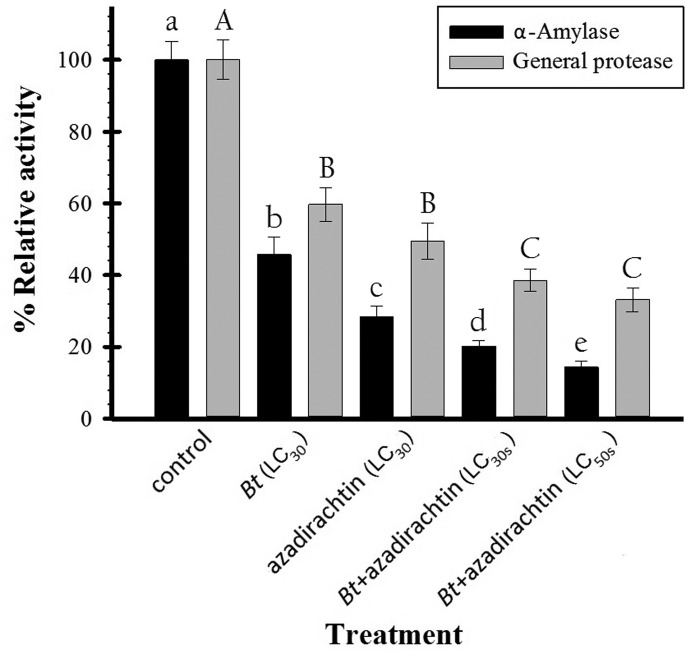
Relative activity (mean ± SE) of digestive enzymes in the midguts of third instar *P. interpunctella* (*n* = 5) that were exposed to *Bt* (LC_30_) or azadirachtin (LC_30_) or both *Bt* and azadirachtin (LC_30_ or LC_50_ concentrations of each insecticide). Each activity is shown relative to the activity (100%) found in control larvae. The error bars indicate the standard error of the mean of three measurements. Letters above the bars indicate statistically significant differences between values (LSD test, *P* < 0.05).

The midguts of third instar *P. interpunctella* that continuously fed on insecticide-containing diet for 5 d showed statistically significant reductions in α-amylase (*F*_4, 20_ = 98.46, *P*  0.001) and general protease (*F*_4, 20_ = 73.67, *P*  0.001) activities in comparison to midguts from control larvae (Fig. 2). Specifically, the midguts from larvae that fed on diet containing an LC_30_ concentration of *Bt* showed only 45 and 59% of the α-amylase and general protease activities, respectively, found in midguts from control larvae. Similarly, midguts from larvae that fed on diet containing an LC_30_ concentration of azadirachtin showed only 28 and 49% of the α-amylase and general protease activities, respectively, found in control midguts. Midguts from larvae that fed on diet containing LC_30_ concentrations of both *Bt* and azadirachtin showed even lower α-amylase and general protease activities (20 and 38%, respectively) in comparison to midguts from control larvae, and also in comparison to midguts from larvae that fed on diet containing only one of the insecticides (Fig. 2). The midguts from larvae that fed on diet containing LC_50_ concentrations of both *Bt* and azadirachtin showed the greatest reductions in α-amylase and general protease activities (14 and 33%, respectively) in comparison to midguts from control larvae, and also in comparison to midguts from larvae that were fed on diet containing only one of the insecticides (Fig. *2*). In addition, the midguts from these larvae showed significantly (*P* < 0.05) lower α-amylase activity in comparison to midguts from larvae that fed on diet containing LC_30_ concentrations of both *Bt* and azadirachtin. A statistically significant difference, however, was not found in general protease activity between these two groups (Fig. 2).

### Nutritional Indices of *P. interpunctella* Following Insecticide Exposure

Third instar *P. interpunctella* that continuously fed on insecticide-containing diet for 5 d showed statistically significant reductions in food consumption (*F*_4, 45_ = 24.97, *P* 0.001) and weight gain (*F*_4, 45_ = 41.79, *P* 0.001) in comparison to control larvae (Table 5). Larvae that fed on diet containing an LC_30_ concentration of *Bt* showed a 22% reduction in diet consumption and 71% reduction in weight gain in comparison to control larvae. Similarly, larvae that fed on diet containing an LC_30_ concentration of azadirachtin showed a 28% reduction in diet consumption and 71% reduction in weight gain in comparison to control larvae. Larvae that fed on diet containing LC_30_ concentrations of both *Bt* and azadirachtin showed a slightly higher reduction in food consumption (31%) and weight gain (79%) in comparison to control larvae. The level of food consumption and weight gain was statistically unchanged between larvae that fed on diet containing LC_30_ or LC_50_ concentrations of both insecticides (Table 5).

**Table 5:** Nutritional indices of third-instar larvae of *P. interpunctella* following exposure to *Bt* and azadirachtin**^*a*^**

Treatment	***n***^*b*^	Food consumed (mg/20 larvae)	Larval weight gain (mg/20 larvae)	AD (%)	CI	ECI (%)	ECD (%)	RCR (mg/mg/d)	RGR (mg/mg/d)
**Control**	**10**	412.7 ± 8.1a	158.7 ± 5.4a	83.3 ± 0.7a	9.6 ± 0.0a	38.4 ± 0.4a	46.1 ± 0.3a	0.96 ± 0.01a	0.368 ± 0.001a
***Bt* (LC_30_)**	**10**	323.1 ± 12.4b	46.2 ± 1.5b	60.9 ± 0.7b	7.8 ± 0.1b	14.3 ± 0.2c	23.5 ± 0.2c	0.78 ± 0.01b	0.111 ± 0.001b
**azadirachtin (LC_30_)**	**10**	296.9 ± 9.8bc	46.8 ± 1.1b	40.8 ± 0.5d	6.7 ± 0.1c	15.8 ± 0.2b	38.1 ± 0.3b	0.67 ± 0.00c	0.106 ± 0.001c
***Bt* + azadirachtin (LC_30_)**	**10**	283.2 ± 13.0c	32.7 ± 1.1c	49.7 ± 0.6c	5.9 ± 0.1d	11.5 ± 0.2d	23.3 ± 0.2c	0.59 ± 0.01d	0.067 ± 0.001d
***Bt* + azadirachtin (LC_50_)**	**10**	273.3 ± 12.2c	31.6 ± 0.7c	49.8 ± 0.8c	5.4 ± 0.1d	11.5 ± 0.1d	23.2 ± 0.2c	0.54 ± 0.01d	0.062 ± 0.001d

^*a*^ The larvae were continuously exposed to LC_30_ (341.7 and 155.9 µg a.i./ml, respectively) and LC_50_ (490.4 and 241.1 µg a.i./ml, respectively) concentrations of *Bt* and azadirachtin for 5 d prior to determining mass. Mean values followed by different letters in the same column are significantly different (LSD, *P* < 0.05).

^*b*^ The n value shows the sample size for each parameter.

AD, approximate digestibility; CI, consumption index; ECI, efficiency of conversion of ingested food; ECD, efficiency of conversion of digested food; RCR,  relative consumption rate; RGR, relative growth rate.

The nutritional indices (AD, CI, ECI, ECD, RCR, and RGR) of third instar *P. interpunctella* that fed on diet containing *Bt* (LC_30_ concentration) or azadirachtin (LC_30_ concentration) or both *Bt* and azadiracthin (LC_30_ or LC_50_ concentrations of each insecticide) were significantly (*F*_4, 45_ = 59.91, *P*  0.001 for AD; *F*_4, 45_ = 29.09, *P*  0.001 for CI; *F*_4, 45_ = 229.98, *P*  0.001 for ECI; *F*_4, 45_ = 157.75, *P*  0.001 for ECD; *F*_4, 45_ = 63.58, *P*  0.001 for RCR and *F*_4, 45_ =  99.01, *P*  0.001 for RGR) lower than those of control larvae (Table 5). In general, larvae that were exposed only to *Bt* (LC_30_ concentration) or azadirachtin (LC_30_ concentration) showed higher nutritional indices than those that were exposed to both *Bt* and azadirachtin (LC_30_ or LC_50_ concentrations of each insecticide). Two exceptions were found in the AD value of larvae that were exposed to azadirachtin and the ECD value of larvae that were exposed to *Bt*. Larvae that were exposed to only azadirachtin (LC_30_ concentration) showed a lower AD value than larvae that were exposed to both *Bt* and azadirachtin (either LC_30_ or LC_50_ concentrations of each insecticide). And, larvae that were exposed to only *Bt* (LC_30_ concentration) showed an ECD value that was statistically identical to ECD values of larvae that were exposed to both *Bt* and azadirachtin (either LC_30_ or LC_50_ concentrations of each insecticide).

## Discussion

The potential development of resistance to *Bt* in lepidopteran pests has prompted researchers to find strategies that will preserve this environmentally friendly insecticide (Zhu et al. 2007). One such strategy is the co-application of azadirachtin with *Bt*. In comparison to most synthetic chemical insecticides; azadirachtin (like *Bt*) has a more environmentally friendly footprint (Hoelmer et al. 1990). However, several factors such as the type of formulation, dosage, type of commodity, target species, temperature, and relative humidity affect its efficacy (Kavallieratos et al. 2007). We report here the efficacy and effects of the application of *Bt* and azadirachtin against larval *P. interpunctella*. Individually, these insecticides induced severe toxicity in larval *P. interpunctella* in a dose-dependent manner. On the basis of the calculated LC_50_ value of azadirachtin alone or *Bt* alone, azadirachtin was about 2-fold more toxic than *Bt*. This difference may result from the nature and mode of action of these insecticides. A similar difference in the LC_50_ values of these insecticides is found in larval *Helicoverpa armigera* Hübner (Lepidoptera: Noctuidae) (Abedi et al. 2014). Larval *P. interpunctella* that fed on diet containing both *Bt* and azadirachtin showed increased mortality, reduced energy reserves, reduced weight, and reduced nutritional indices. Exposure to LC_30_ concentrations of both *Bt* and azadirachtin resulted in an additive interaction in terms of mortality. This interaction was more pronounced when the larvae fed on diet containing LC_50_ concentrations of each insecticide; in this case the interaction was synergistic. Singh et al. (2007) found similar positive interactions in larval *H. armigera* that were exposed to both *Bt* and azadirachtin.

Rharrabe et al. (2008) show that energy reserves are reduced in larval *P. interpunctella* that are reared on diet incorporated with azadirachtin. We found that energy reserves (i.e., total protein, glycogen, and lipid content) were reduced to a greater extent in larvae that fed on diet containing both *Bt* and azadiracthin. We hypothesize that this reduction results from the antifeedant activity of *Bt* and azadirachtin as well as from a reduction in food digestion caused by the toxic effects of these insecticides. These potential toxic effects include the reduced activity of digestive enzymes such as α-amylase and general proteases. The reduction in digestive enzyme activity may result from cytotoxic effects of *Bt* and azadirachtin on midgut epithelial cells which synthesize these enzymes or possibly from the direct inhibition of these enzyme. Railda Roel et al. (2010) shows that the ingestion of azadirachtin extracts induce significant cytotoxic effects on midgut epithelial cells. Zhu et al. (2007) also report cytotoxic effects of *Bt* toxins when they bind to receptors on midgut epithelium cells resulting in the disruption of gut function. The synergism shown by *Bt* and azadirachtin may be partially explained by their binding to different receptors on the same midgut cell and targeting of the same tissue. Studies abound about interaction of *Bt* and other biological control factors. For instance, The synergistic effects between *Bt* and azadirachtin and between *Bt* and an extract from *Vitex pseudo-negundo* (Hausskn) (Verbenaceae) was investigated on the rice leafroller *Cnaphalocrocis medinalis* (Guenée) (Lepidoptera: Pyralidae) (Mancebo et al. 2002).

In the long-term, larvae that feed on low-quality diet will show reduced feeding whereas larvae that feed on high-quality diet will show increased feeding (Hemati et al. 2012, Borzoui et al. 2015). In this study, the lowest amount of food consumed was observed in larvae that were reared on diet containing azadirachtin. As discussed above, this reduction in feeding is at least partially attributed to a reduction in α-amylase activity. Since *P. interpunctella* feeds on polysaccharide-rich diets, the level of α-amylase may be a key factor in the efficient propagation of this pest insect (Mendiola-Olaya et al. 2000).

Body weight is a primary fitness index when characterizing insect population dynamics (Liu et al., 2004). Larval *P. interpunctella* that were reared on insecticide treated diet showed variation in nutritional indices, in particular ECI and ECD values, that strongly correlated to larval mass, the amount of food consumed, and the mass of feces produced. These finding suggest that both *Bt* and azadirachtin induce antifeedant activity against larval *P. interpunctella*. Larvae that fed on *Bt* and azadirachtin treated diet showed decreased α-amylase and general protease activities in comparison to larvae that fed on control diet. Similar findings are reported by Walker et al. (1998) suggesting that the inhibition of these digestive enzymes by these insecticides results in the reduction of CI, ECI, and ECD.

The highest RCR value for control larvae demonstrated that the weight of food consumed relative to the mean weight gain of larvae over the feeding period was the highest for these larvae. Also, the RGR value was the highest for larval *P. interpunctella* that were reared on control diet and the lowest for those that were reared on diet containing both *Bt* and azadirachtin. This demonstrated that larvae that fed on control diet had higher efficiency than larvae fed on diet containing both *Bt* and azadirachtin in terms of the conversion of ingested food into body mass. The ability of *Bt* and azadirachtin to reduce the RCR, ECI, RGR, approximate digestibility, and assimilation rate of food has been previously observed (Railda Roel et al. 2010; Shannag et al. 2015).

In conclusion, *Bt* and azadirachtin induce significant insecticidal effects on third-instar larvae of *P. interpunctella*. Higher mortality was observed when the larvae fed on diet containing both *Bt* and azadirachtin in comparison to larvae that fed on diet containing only one of these insecticides. *Bt* and azadirachtin significantly reduced the activity of amylolytic and proteolytic enzymes, food consumption, larval weight gain, and nutritional efficiency. Exposure to a combination of *Bt* and azadirachtin led to a greater reduction of these parameters. We suggest that the use of azadirachtin as an additive to a *Bt* product may result in a more effective management strategy for *P. interpunctella* and related pest insects. Unlike the most chemical insecticides, there were no indications of any long-term effects of exposure to environmentally insecticides in the population dynamics of the natural enemy community (Naranjo, 2005). So, these selective pest control technology and combining environmentally benign insecticides that negatively affect the fitness of the target pest should broaden opportunities for natural enemy conservation in store.

## Acknowledgments

The work received financial support by the University of Mohaghegh Ardabili which is greatly appreciated.
